# 代谢组学方法分析肺癌患者血清和尿液小分子代谢产物的初步研究

**DOI:** 10.3779/j.issn.1009-3419.2012.04.01

**Published:** 2012-04-20

**Authors:** 艳洁 牛, 银玲 江, 长江 许, 向迎 王, 友如 刘, 珩 赵, 宝惠 韩, 丽岩 姜

**Affiliations:** 1 200030 上海，上海交通大学附属胸科医院肺内科 Department of Pulmonology, Chest Hospital Affiliated to Jiaotong University, Shanghai 200030, China; 2 201203 上海，上海中药创新研究中心 Shanghai Innovative Research Center of Traditional Chinese Medicine, Shanghai 201203, China; 3 200030 上海，上海交通大学附属胸科医院胸外科 Department of Thoracic Surgery, Chest Hospital Affiliated to Jiaotong University, Shanghai 200030, China

**Keywords:** 肺肿瘤, 代谢组学, 气相色谱/质谱, 诊断, 生物学标志物, Lung neoplasms, Metabolomics, Gas chromatography/Mass spectrometry, Diagnosis, Biological markers

## Abstract

**背景与目的:**

肺癌是当今世界各国最常见的恶性肿瘤之一。目前尚没有寻找到理想的用于肺癌诊断的肿瘤标志物，因而尝试用各种新方法来探索新的生物学标志物已成为肺癌研究的热点。本研究采用代谢组学技术对肺癌患者和其它肺部疾病患者血清及尿液中的小分子代谢物质进行分析，以寻求潜在的肺癌肿瘤标志物。

**方法:**

运用气相色谱/质谱法（gas chromatography/mass spectrometry, GC/MS）对19例肺癌与15例其它肺部疾病患者的血清及尿液样本进行代谢组学分析，采用正交偏最小二乘判别分析法（orthogonal to partial least squares discriminant analysis, OPLS-DA）进行建模，运用两样本的t检验寻找两组间差异性代谢产物。

**结果:**

检测到血清中代谢产物共57种，尿液中代谢产物共38种，多变量统计结果显示肺癌患者与其它肺部疾病患者的代谢谱有明显差异，根据*t*检验结果寻找到血清相关的差异代谢产物13种，尿液相关的差异代谢产物7种。

**结论:**

利用代谢组学方法能区分肺癌与其它肺部疾病患者，其结果在分子水平辅助肺癌的诊断、未来作为新技术应用于肺癌的诊断有一定的前景。

## Abstract (en)

**Background and objective:**

Lung cancer is one of the most common cancers worldwide. Thus far, good tumor markers for diagnosing this disease have not been found. Therefore, the discovery of novel biomarkers through the application of new methods has become a hotspot in lung cancer research. The aim of this study is to analyze low-molecular-weight metabolites in the serum and urine samples of lung cancer patients and patients with other lung diseases through metabolomics and to explore potential tumor markers further.

**Methods:**

Both serum and urine samples from 19 lung cancer patients and 15 patients with other lung diseases were subjected to metabolomic analysis using gas chromatography mass spectrometry. Orthogonal to partial least squares discriminant analysis was performed for modeling. Two sample t-test was used to identify differences in metabolite concentrations.

**Results:**

A total of 57 metabolites were found in the serum, and 38 metabolites were found in the urine. Multivariate statistical analysis yielded a significant distinction in the metabolic profiles between lung cancer patients and patients with other lung diseases. The *t*-test results indicated a total of 13 metabolites in the serum and 7 metabolites in the urine with statistically significant differences.

**Conclusion:**

Metabolomics is useful in discriminating between lung cancer and other lung diseases. As a novel approach, it has potential in the diagnosis of lung cancer at molecular level.

肺癌是当今世界各国最常见的恶性肿瘤之一。2008年全球统计数据显示肺癌在所有癌症新发病例和死亡病例中分别占13%和18%，已居男性癌症发病率和死亡率首位，在女性癌症死因中也居第2位^[1]^。我国的回顾性调查研究^[2]^发现，近40年来肺癌在癌症死亡中的构成也由7.35%上升至22.70%，从第5位的癌症死因跃居第1位。由于肺癌起病隐匿，大多数患者就诊时已属晚期，失去手术治疗的机会，而临床诊断Ⅰa期与Ⅳ期肺癌患者的5年生存率从50%下降到2%^[3]^，因此早期诊断是降低肺癌死亡率的重要措施。随着分子生物学的发展，肿瘤标志物对恶性肿瘤的筛查有重要的临床价值，而现有的传统肿瘤标志物对于肺癌的诊断尚缺乏特异性与灵敏度。

代谢组学是对某一生物或细胞所有小分子量代谢产物进行定性和定量分析的一门新兴学科，其揭示的小分子代谢产物变化是机体内基因、蛋白质/酶等功能变化的一系列事件的最终结果，直接反映了生物体系的最终状态，可以反映机体特定病理生理状态下整体代谢物质的变化，为疾病的诊断提供了新的研究思路^[4]^。目前随着代谢组学技术的不断发展，越来越多的肿瘤相关代谢标志物在肿瘤疾病中被鉴定^[5]^，相关的研究主要集中在乳腺癌^[6]^、卵巢癌^[7]^、前列腺癌^[8]^等方面。而针对肺癌的代谢组学研究处于起步阶段，尚未有应用代谢组学方法检测肺癌患者血清和尿液小分子代谢产物的联合分析报道。本研究通过气相色谱/质谱(gas chromatography/mass spectrometry, GC/MS)化学衍生法分析初治肺癌与其它肺部疾病患者血清和尿液的小分子代谢产物，以探讨代谢组学方法在肺癌诊断中的应用。

## 1 材料与方法

### 1.1 研究对象

病理或细胞学证实的19例肺癌患者作为肺癌组，15例其它肺部疾病患者作为对照组。肺癌组年龄分布31岁-71岁，男性17例、女性2例；腺癌10例、鳞癌6例、小细胞肺癌3例；根据国际抗癌联盟(Union for International Cancer Control, UICC)第七版肺癌TNM分期，Ⅰ期、Ⅱ期、Ⅲ期、Ⅳ期患者分别为3例、5例、7例、4例；有吸烟史患者11例。对照组年龄分布18岁-68岁，男性9例、女性6例；肺炎5例、支气管扩张3例、肺结核5例、肺错构瘤1例、肺淋巴瘤1例；有吸烟史患者6例。由于代谢产物受多种因素的影响，如患者饮食习惯、合并症、药物使用情况等，为了尽量减少各种其它因素对研究结果的影响，所有入组病例都作以下筛选：①均为非素食者；②无代谢性疾病和长期服药史；③肺癌患者入组前未接受任何抗肿瘤治疗，体力状态(performance status, PS)评分 < 2分。入组前均签署知情同意书。

### 1.2 样本采集

收集入选患者空腹血标本4 mL于无菌促凝BD真空采血管中，2, 000 rpm(台式大容量冷冻离心机TDL-5M)、4 ℃离心5 min，取上层血清，与同时留取的尿液5 mL共同保存于-80 ℃冰箱中待用。

### 1.3 血清样本预处理

将低温保存的血清样本放置室温解冻摇匀，取100 µL加入内标十七酸10 µL混匀。再加入甲醇：氯仿(3:1)混合溶液300 µL，振荡30 s，置于-20 ℃冰箱中10 min。以10, 000 rpm(高速冷冻离心机GL21M，KAIDA)离心10 min，取上清液300 µL加入进样瓶，室温N2吹干后加入甲氧胺吡啶溶液80 µL，振荡30 s，置于30 ℃摇床以200 rpm(恒温振荡器HZ-9201K)反应90 min。最后加入80 µL硅烷化试剂[Bis-(trimethylsilyl)trifluoroacetamide, BSTFA]+1%三甲基硅烷(Trimethylsilyl Compounds, TMCS)，振荡30 s，在70 ℃烘箱反应60 min，振荡30 s。室温放置1 h后准备进样。

### 1.4 尿液样本预处理

将低温保存的尿液样本在室温解冻摇匀，取1 mL以12, 000 rpm(高速冷冻离心机GL21M，KAIDA)离心10 min后取上清200 µL，加入30 U尿素酶，振荡30 s后37 ℃水浴15 min去除尿素；再加入800 µL甲醇，10 µL内标十七酸，振荡1 min，13, 000 rpm离心10 min，取上清液200 µL加入进样瓶，室温N2吹干后加入50 µL甲氧胺吡啶溶液，振荡1 min，置于30 ℃摇床以200 rpm(恒温振荡器HZ-9201K)反应90 min，然后加入50 µL BSTFA+1%TMCS，振荡30 s，在70 ℃烘箱反应60 min，振荡30 s，最后加入40 µL庚烷，振荡30 s，准备进样。

### 1.5 气相色谱/质谱(GC/MS)分析条件

气相色谱条件：系统载气氦气，流速1 mL/min。血清检测色谱柱升温程序：起始温度80 ℃保持2 min，以10 ℃/min升温至180 ℃后即刻以5 ℃/min升温至240 ℃，再以25 ℃/min升温至290 ℃保持9 min，进样口温度270 ℃，接口温度260 ℃。尿液检测色谱柱升温程序：起始温度70 ℃保持2 min，以10 ℃/min升温至170 ℃后即刻以5 ℃/min升温至240 ℃，再以20 ℃/min升温至300 ℃保持6 min，进样口温度260 ℃，接口温度270 ℃。质谱条件：溶剂延时5 min，离子源温度200 ℃，电子轰击电离，电子能量70 eV，全扫描范围(m/z)30-600。取预处理后血清或尿液样品1 µL进样。

### 1.6 数据处理和统计

记录每个血清及尿液样本的总离子流色谱图(total ions chromatogram, TIC)，将TIC导入美国国家标准与技术研究院(National Institute of Standards Technology, NIST)8.0质谱数据库中，逐个分析每个样本的总离子流色谱图的每个峰，在NIST 8.0质谱库中检索相应的化合物，选择匹配度较好的化合物。以代谢物峰面积与内标十七酸峰面积比值来标准化数据，将GC/MS分析得到的数据转换成通用数据格式(common data format, CDF)，并进行峰数据提取，得到质荷比、保留时间和谱峰编号的三维信息，再将此信息导入SIMCA-P 12.0+软件中进行多变量统计分析，采用监督的正交偏最小二乘判别分析法(orthogonal to partial least squares discriminant analysis, OPLS-DA)进行建模分析。运用两样本的t检验比较肺癌组和其它肺部疾病组的代谢产物水平。*P* < 0.05为差异有统计学意义，寻找差异性代谢产物。以肺癌组与其它肺部疾病组的比值表示该物质在肺癌患者的相对水平， > 1表示该物质在肺癌患者中升高， < 1表示该物质在肺癌患者中降低。

## 2 结果

### 2.1 血清GC/MS检测分析结果

根据NIST 8.0质谱数据库分析鉴定血清样本的TIC，得到匹配度较好的代谢物共57种(表 1)，OPLS-DA法分析肺癌组和其它肺部疾病组患者血清样本的二维分布结果(图 1A)所示肺癌组主要位于第1、4象限，其它肺部疾病组主要位于第2、3象限，可见两组有明显的分离趋势，并根据t检验结果鉴定得到13种差异性代谢产物(表 2)。

**1:** 肺癌及其它肺部疾病患者血清质谱峰保留时间，物质名称及符合度列表 Serum metabolites of lung cancer and other lung diseases

Retention time (min)	Metabolites	Match percent (%)
5.351	Acetic acid	82
5.787	Pyruvate	89
5.815	Lactate	90
5.997	2-Hydroxy butanate	90
6.096	2-Hydroxybutyric	92
6.108	3-Hydroxy butanate	87
6.175	Pentanoate	98
6.387	Ethanedioic acid	84
6.636	Alanine	86
6.919	Valine	94
7.018	Testosterone	78
7.383	Glyceral	82
7.671	Xylitol	80
8.666	Urea	94
9.153	Leucine	91
9.226	Phosphric acid	98
9.472	Isoleucine	87
9.529	Proline	95
9.661	Glycine	91
10.121	Amber acid	97
10.405	Serine	83
10.775	Threonine	91
11.065	Creatinine	83
11.354	Dimethoxyphenylethylamine	72
11.592	Malonic acid	83
12.258	Aminomalonic acid	95
12.563	Propionic acid	91
13.931	Phenylalanine	91
14.142	Xylose	82
14.238	Maleic acid	82
14.375	Propylamine	81
14.685	Aspartic acid	83
16.034	Glutaminate	87
16.739	Ornithine	96
16.835	Citrate	87
17.014	Ribitol	78
17.378	Galctofuranose	78
18.037	Glucose	95
18.254	Mannose	90
18.366	Lysine	90
18.762	Tyrosine	91
18.962	Gulonic acid	90
19.161	Nuceic acid	99
19.546	Glucopyranose	94
20.146	Hexadecanoic acid	98
21.371	Cyclohexanhexol	90
21.497	Uric acid	93
21.741	Heptadecanoic acid	95
22.650	Tryptophane	90
22.816	9, 12-Octadecadienoic acid	81
23.154	Trans-9-Octadecenoic acid	80
23.342	Octadecanoic acid	91
25.485	Arachidonic acid	93
29.473	Glucitol	83
32.282	VitaminE	95
32.567	Benzoic acid	85
33.089	Cholesterol	96

**1:**
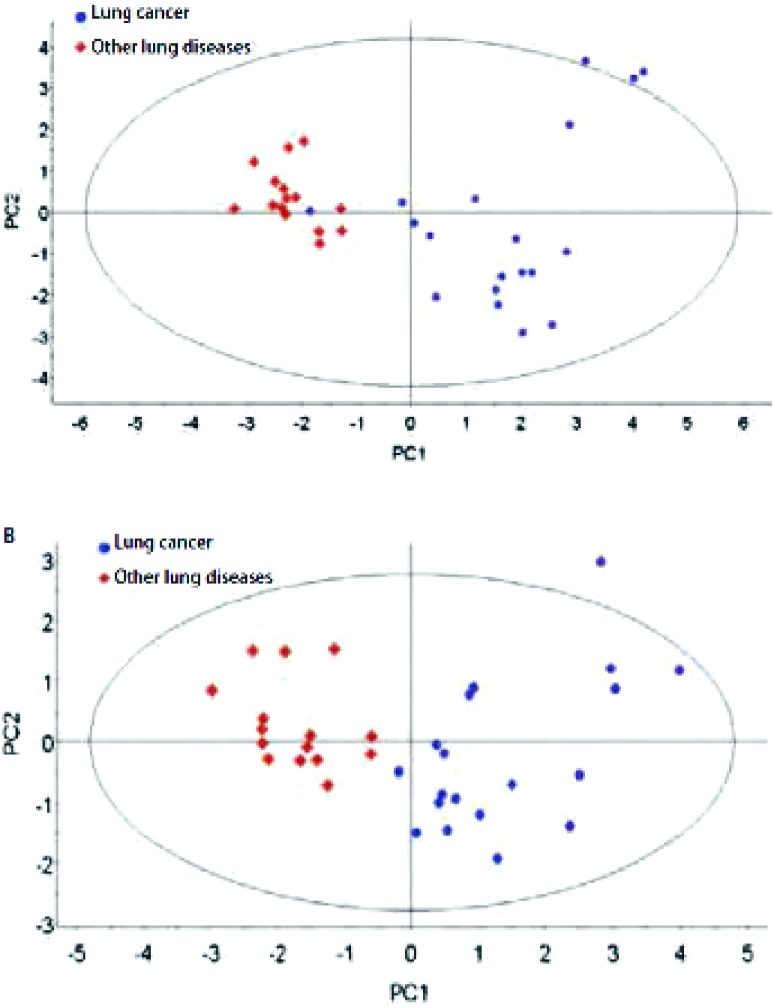
肺癌组与其它肺部疾病组血清样本（A）和尿液样本（B）OPLS-DA代谢模式识别图。蓝色圆点代表肺癌患者，红色菱形代表其它肺部疾病患者。 OPLS-DA scores plot discriminating serum (A) and urine (B) samples of lung cancer patients and patients with other lung diseases based on the metabolite profiling data. Blue circles indicate lung cancer patients; red diamonds indicate patients with other lung diseases.

**2:** 肺癌患者组和其它肺部疾病组间的血清差异性代谢物 Significantly changed metabolites in serum of lung cancer and other lung diseases

Metabolites	P^*^	Tendency	Ratio (lung cancer/other lung diseases)
Pyruvate	< 0.000, 001	↑	1.86
Lactate	0.000, 099	↑	1.54
Alanine	0.001, 044	↑	1.44
2-Hydroxy butanate	0.000, 007	↑	2.38
Ethanedioic acid	0.000, 081	↑	1.85
3-Hydroxy butanate	0.000, 007	↑	1.84
Glyceral	0.000, 302	↑	1.78
Proline	0.000, 189	↑	1.55
Ribitol	0.013, 100	↓	0.91
Citrate	0.000, 445	↑	2.00
Lysine	0.002, 043	↓	0.69
Hexadecanoic acid	0.030, 245	↑	1.39
9, 12-Octadecadienoic acid	0.000, 008	↑	1.69
^*^Statistical *P*-value calculated using a two sample *t*-test (significance at *P* < 0.05); ↑: The level of the metabolites is higher in lung cancer than in other lung diseases; ↓: The level of the metabolites is lower in lung cancer than in other lung diseases.			

### 2.2 尿液GC/MS检测分析结果

根据NIST 8.0质谱数据库分析鉴定尿液样本的TIC，得到匹配度较好的代谢物共38种(表 3)，OPLS-DA法分析肺癌组和其它肺部疾病组患者尿液样本的二维分布结果(图 1B)所示肺癌组主要位于第1、4象限，其它肺部疾病组主要位于第2、3象限，可见两组有明显的分离趋势，并根据*t*检验结果鉴定得到7种差异性代谢产物(表 4)。

**3:** 肺癌及其它肺部疾病患者尿液质谱峰保留时间、物质名称及符合度列表 Urine metabolites of lung cancer and other lung diseases

Retention time (min)	Metabolites	Match percent (%)
5.806	Acetic acid	91
6.221	Alanine	80
7.800	Hydroxyisovaleric acid	99
8.326	Urea	86
8.817	Phosphric acid	98
9.213	Glycine	83
9.786	Hydroxygamma-butyrolactone	74
9.990	Serine	91
10.353	Threonine	90
10.873	Tetramethylenediamine	86
11.658	Aminolevulinic acid	72
11.961	Butane	90
12.027	Proline	94
12.475	Creatinine	99
12.626	3-Hydroxy butanate	94
12.857	Dithiane	97
13.483	Phenylacetic acid	96
14.267	Butyl aldehyde	81
14.338	Maleic acid	79
14.525	Propylamine	77
14.715	Xylitol	93
14.887	Arabitol	95
15.105	Acroleic	80
15.586	Ribotide	76
15.872	Ornithine	84
16.343	Citrate	90
17.417	Mannitic acid	70
17.727	Mannose	91
18.004	Glucose	91
18.228	Lactose	86
19.427	Glucanic acid	83
19.612	Hexadecanoic acid	99
21.061	Uric acid	90
20.831	Inositol	81
21.219	Heptadecanoic acid	97
21.564	Trans-9-Octadecenoic acid	83
22.835	Octadecanoic acid	97
29.128	Glucopyranose	95

**4:** 肺癌患者组和其它肺部疾病组间的尿液差异性代谢物 Significantly changed metabolites in urine of lung cancer and other lung diseases

Metabolites	P^*^	Tendency	Ratio（lung cancer/other lung diseases）
Creatinine	0.000, 055	↑	1.71
Inositol	0.005, 763	↑	1.49
3-Hydroxy butanate	0.004, 642	↑	1.70
Ribotide	0.000, 594	↓	0.75
Citrate	0.000, 015	↑	2.11
Hexadecanoic acid	0.000, 365	↑	2.24
Trans-9-Octadecenoic acid	0.009, 524	↑	1.60
^*^Statistical *P*-value calculated using a two sample *t*-test (significance at *P* < 0.05); ↑: The level of the metabolites is higher in lung cancer than in other lung diseases; ↓: The level of the metabolites is lower in lung cancer than in other lung diseases.			

## 3 讨论

目前己知的肺癌肿瘤标志物主要有激素、胚胎蛋白、细胞表面膜抗原、酶类及某些细胞因子等^[9]^，临床应用比较广泛的有癌胚抗原(carcinoembryonic antigen, CEA)、神经元特异性烯醇化酶(neuron specific enolase, NSE)、细胞角蛋白19片断抗原(cytokeratin fragment antigen 21-1, CYFRA21-1)及鳞状细胞癌相关抗原等。单一标志物对肺癌的诊断价值有限，目前已倾向多种标志物联合检测的方法，以提高肺癌诊断的特异性和敏感性^[10]^。但与肺癌有关的众多标志物在肺癌诊断的敏感性及特异性方面仍不令人满意，尝试用各种新方法来探索肺癌相关的特异性分子标志物已经成为研究热点。

代谢组学是继基因组、转录组、蛋白质组之后，应运而生并在近几年蓬勃发展，逐步应用于医学领域的新兴学科，它利用高通量的分离与检测技术尽可能多的识别样品中的小分子代谢物质，是一种全面反映机体各个器官功能状态的检测方法。随着衍生化试剂和方法的创新，GC/MS开始广泛运用于代谢组学，并逐渐趋于成熟^[11]^。本研究通过应用该技术，分析肺癌和其它肺部疾病患者的血清及尿液小分子代谢产物，采用OPLS-DA建模，发现肺癌患者血清及尿液的代谢模式都出现明显的分离趋势，提示肺癌患者与其它肺部疾病患者由于在不同的病理状态表现出的代谢谱特征有明显差异。本研究结果为今后扩大样本数量进一步验证代谢组学方法在肺癌诊断方面的准确性以及分析不同分期及病理类型肺癌患者代谢谱特征奠定了基础，从而将代谢组学技术逐步引入肺癌的诊断技术领域，为肺癌的诊断探索新的研究方向。

代谢组学方法检测出的一系列小分子代谢产物是机体在特定环境下(如病理状态或药物治疗后)，经过复杂的生化反应后在血液或尿液表现出来的最终结果，其在肺癌的诊断应用有别于通常概念的肿瘤标志物，建立一个有特殊鉴别能力的小分子代谢物群组是代谢组学方法主要的检测目标。本研究发现的13种血清差异代谢产物分别为：乳酸、丙酮酸、柠檬酸、丙氨酸、赖氨酸、脯氨酸、2-羟基丁酸、3-羟基丁酸、乙二酸、十六酸、亚油酸、甘油和核糖醇，其中只有赖氨酸与核糖醇在肺癌组下降，其余11种物质在肺癌组水平均高于其它肺部疾病组。7种尿液差异代谢产物分别为肌酐、肌醇、3-羟基丁酸、核苷酸、柠檬酸、十六酸、油酸，除了核苷酸在肺癌组下降外，其余6种物质在肺癌组水平均高于其它肺部疾病组。这些物质中有些在相关研究被发现并提示了与肿瘤的关系，如乳酸、丙酮酸的增高可能是由于肿瘤组织中Warburg效应的存在^[12]^，是肿瘤细胞能量代谢异常即糖酵解增强的结果；丙氨酸的增高与细胞膜合成代谢增强有关，这在多项前列腺癌的研究^[13, 14]^中均有发现；十六酸、亚油酸、油酸作为细胞膜的重要组成成分，其含量水平与细胞增殖、坏死和凋亡紧密相关，目前已在多种类型肿瘤中发现其水平的改变，如脑肿瘤^[15]^、肉瘤^[16]^、前列腺癌^[17]^等；肌醇是脂肪代谢的前体，主要起调节渗透压、营养细胞、抗氧化及生成表面活性物质的作用，因此细胞增殖加速时增高，多见于星形胶质细胞，在神经胶质瘤中可见增高^[18]^；核苷酸是组成脱氧核糖核酸(deoxyribonucleic acid, DNA)和核糖核酸(ribonucleic acid, RNA)的重要物质，在脂肪酸和糖代谢中是一个关键的中间代谢物，在三磷酸腺苷(adenosine triphosphate, ATP)浓聚物中的改变是肿瘤活力状态的重要指示物。本研究发现肺癌患者尿液中核苷酸含量低于其它肺部疾病患者，提示可能与肿瘤生长消耗大量核苷酸有关。虽然还有一些物质尚未在研究报道中明确与肿瘤的关系，但作为代谢群组中有意义的产物，仍能应用于肺癌的诊断，且随着生物学技术的不断发展及肿瘤基础研究的深入，更多的物质将被揭示出与肿瘤的关系，从而为肿瘤的诊断与治疗提供更宽广的研究思路。代谢组学方法在分子水平辅助诊断肺癌，未来作为新技术应用于肺癌的诊断有一定的前景。
